# Prospects for the Use of Whey for Polyhydroxyalkanoate (PHA) Production

**DOI:** 10.3389/fmicb.2019.00992

**Published:** 2019-05-09

**Authors:** Tiago M. M. M. Amaro, Davide Rosa, Giuseppe Comi, Lucilla Iacumin

**Affiliations:** Dipartimento di Scienze Agroalimentari, Ambientali e Animali, Università degli Studi di Udine, Udine, Italy

**Keywords:** whey, PHA, PHB, polyhydroxyalkanoate, bioplastics, environment, lactose

## Abstract

Plastic production and accumulation have devastating environmental effects, and consequently, the world is in need of environmentally friendly plastic substitutes. In this context, polyhydroxyalkanoates (PHAs) appear to be true alternatives to common plastics because they are biodegradable and biocompatible and can be biologically produced. Despite having comparable characteristics to common plastics, extensive PHA use is still hampered by its high production cost. PHAs are bacterial produced, and one of the major costs associated with their production derives from the carbon source used for bacterial fermentation. Thus, several industrial waste streams have been studied as candidate carbon sources for bacterial PHA production, including whey, an environmental contaminant by-product from the dairy industry. The use of whey for PHA production could transform PHA production into a less costly and more environmentally friendly process. However, the efficient use of whey as a carbon source for PHA production is still hindered by numerous issues, including whey pre-treatments and PHA producing strain choice. In this review, current knowledge on using whey for PHA production were summarized and new ways to overcome the challenges associated with this production process were proposed.

## Introduction

The production of plastics, which are essential in all modern economies, is mostly achieved using fossil feedstocks that have high environmental implications. Moreover, worldwide production of plastics is drastically increasing (from 204 million tons in 2002 to 335 million tons in 2016) and is predicted to continue to increase (PlasticsEurope, 2015, 2017). Production of plastics has led to environmental concerns because plastics are not easily degradable and accumulate in the most varied environments (Barnes et al., 2009). Furthermore, despite remarkable efforts to increase recycling rates (in Europe, recycling increased by 79% from 2006 to 2016), only 31.1% of plastics were recycled in Europe in 2016. The remaining plastics are either disposed of in landfills (27.3%) or used for energy recovery (production of electricity or as fuel for industrial processes) (41.6%) (European Bioplastics)^1^. Consequently, these “conventional” plastics urgently need to be substituted by biologically produced and/or biodegradable plastics, which are called bioplastics. However, even though it is thought that approximately 85% of the plastic products can be substituted by bioplastics, only approximately 1% of the plastics produced in the world are bioplastics (European Bioplastics)^1^. The production of bioplastics is predicted to grow, reaching a production of 2.44 million tons in 2022, of which only 1.086 million tons will be biodegradable (European Bioplastics)^2^. However, this predicted increase is still unsatisfactory compared to the ever-growing global plastic demand (PlasticsEurope, 2017). Polyhydroxyalkanoates (PHAs) are biologically produced polyesters that have been considered extremely valuable alternatives to commonly used petroleum-derived plastics (Madison and Huisman, 1999; Sudesh et al., 2000; Reddy et al., 2003; Verlinden et al., 2007; Akaraonye et al., 2010; Laycock et al., 2014; Li et al., 2016; Mozejko-Ciesielska and Kiewisz, 2016; Dietrich et al., 2017). These molecules accumulate in granules inside bacterial cells and, among other recently reported functions (Koller et al., 2017), provide carbon storage and reducing equivalents (Madison and Huisman, 1999). PHAs possess characteristics similar to common plastics and are biocompatible and biodegradable (Sudesh et al., 2000; Castilho et al., 2009; Laycock et al., 2014; Anjum et al., 2016). Therefore, PHAs are currently being industrially produced and have been used in a broad spectrum of end products, ranging from packaging to medical applications (Akaraonye et al., 2010; Carletto, 2014; Anjum et al., 2016; Mozejko-Ciesielska and Kiewisz, 2016; Dietrich et al., 2017).

Regardless of their excellent features and environmental advantages, more extensive use of PHAs is impaired by their high production costs (Byrom, 1987; Lee, 1996; Reddy et al., 2003; Koller et al., 2007a). One of the main factors contributing to these high costs is the use of expensive fermentation carbon sources, which have been shown to account for approximately 40% of the total PHA production costs (Choi and Lee, 1999; Kim, 2000). Thus, the use of waste materials as carbon sources for microbial-derived PHA production has been proposed in order to simultaneously reduce both PHA production and waste disposable costs (Choi and Lee, 1999; Kim, 2000; Koller et al., 2017; Nielsen et al., 2017). Several waste sources have been used to produce PHAs with relative success (Marshall et al., 2013; Nikodinovic-Runic et al., 2013; Anjum et al., 2016; Koller et al., 2017), including domestic wastewater (Carucci et al., 2001); food waste (Rhu et al., 2003); molasses (Albuquerque et al., 2007; Carvalho et al., 2014); olive oil mill effluents (Dionisi et al., 2005); palm oil mill effluents (Din et al., 2012); tomato cannery water (Liu et al., 2008); lignocellulosic biomass (Bhatia et al., 2019); coffee waste (Bhatia et al., 2018); starch (Bhatia et al., 2015); biodiesel industry waste (Kumar et al., 2014a; Sathiyanarayanan et al., 2017); used cooking oil (Ciesielski et al., 2015; Kourmentza et al., 2017); pea-shells (Patel et al., 2012; Kumar et al., 2014b); paper mill wastewater (Jiang et al., 2012); bio-oil from the fast-pyrolysis of chicken beds (Moita and Lemos, 2012); and cheese whey (Table 1).

**Table 1:** Summary of the studies aimed at producing PHAs from whey divided by the whey pre-treatment type.

Substrate	Type of culture	Microorganism	Culture method	Productivity^1^	Type of PHA	Reference
Whey powder supernatant + additives	Pure cultures	*Alcaligenes latus*	Batch culture	0.11 g/L/h	PHB	Berwig et al., 2016
	Engineered cultures	Engineered *Escherichia coli* with *Alcaligenes latus* PHA biosynthesis genes	Fed-batch culture	2.57 g/L/h; 4.6 g/L/h; 1.35 g/L/h	PHB	Ahn et al., 2000, 2001; Park et al., 2002
		Engineered *Escherichia coli* with *Cupriavidus necator* PHA biosynthesis genes	Batch culture; Fed-batch culture; Fed-batch culture; Fed-batch culture; Fed-batch culture	5.2 g/L/h; 1.408 g/L/h; 0.90 g/L/h; 0.57 g/L/h; 0.33 g/L/h	PHB	Lee et al., 1997; Wong and Lee, 1998; Kim, 2000; Farinha, 2009; Pais et al., 2014
		Engineered *Escherichia coli* with *Azotobacter* sp. PHA biosynthesis genes	Fed-batch culture	2.13 g/L/h	PHB	Nikel et al., 2006
	MMC	Undefined	Batch culture	0.0035 g/L/h; 0.0226 g/L/h	Not specified; PHB	Bosco and Chiampo, 2010; Carletto, 2014
Whey permeate + additives	Pure cultures	*Sinorhizobium meliloti*; *Bacillus megaterium*; *Bacillus* spp*.; Sinorhizobium* spp.	Batch culture	0.005 g/L/h	PHB	Povolo and Casella, 2003
		*Hydrogenophaga pseudoflava*	Batch culture	0.0039 g/L/h; 0.05 g/L/h	PHB;P-3(HB-co-HV)	Povolo and Casella, 2003; Koller et al., 2007b
		*Methylobacterium* sp. *ZP24*	Batch culture	0.02 g/L/h	PHB	Yellore and Desai, 1998
	Engineered cultures	Engineered *Cupriavidus necator* with *Escherichia coli* lactose degradation genes	Batch culture	0.02 g/L/h	PHB	Povolo et al., 2010
	MMC	Undefined	Batch culture	0.018 g/L/h	PHB	Carletto, 2014
Hydrolyzed whey permeate + additives	Pure cultures	*Pseudomonas hydrogenovora*	Fed-batch culture; Batch culture; Fed-batch culture	NS;0.03 g/L/h; 0.04 g/L/h	PHB;PHB;P-3(HB-co-HV);	Koller et al., 2006, 2007b, 2008
		*Haloferax mediterranei*	Fed-batch culture; Fed-batch culture; Batch culture; Fed-batch culture	NS; 0.14 g/L/h; 0.05 g/L/h; 0.148 g/L/h	P(3HB-co-3HV-co-4HB);P-(3HB-co-3HV)/P(3HB-co-3HV-co-4HB);P-3(HB-co-HV);P-(3HB-co-3HV)	Koller et al., 2006, 2007a,b; Koller, 2015
		*Cupriavidus necator*	Fed-batch culture	0.17 g/L/h	P-(3HB-co-3HV)	Marangoni et al., 2002
		*Hydrogenophaga pseudoflava*	Batch culture	0.012 g/L/h	P-3(HB-co-HV)	Koller et al., 2007b
	Engineered cultures	Engineered *Cupriavidus necator* with *Escherichia coli* lactose degradation genes	Batch culture	0.7 g/L/h	PHB	Povolo et al., 2010
Whey + additives	Pure cultures	*Bacillus cereus PS-10*	Batch culture	NS	PHB	Sharma and Bajaj, 2015
		*Pseudomonas aeruginosa*	Batch culture	0.005 g/L/h	Diverse	Singh and Mallick, 2009
Fermented whey powder permeate	MMC	Undefined	Fed-batch culture; Fed-batch culture; Batch culture; Fed-Batch culture	0.56 g/L/h; 0.25 g/L/h; 0.80 Cmol/Cmol; 0.204 g/L/h	P-3(HB-co-HV)	Duque et al., 2014; Oliveira et al., 2017; Carvalho et al., 2018; Domingos et al., 2018
Whey supernatant + additives	Pure cultures	*Bacillus megaterium*	Batch culture	0.06 g/L/h	PHB	Obruca et al., 2011
		*Thermus thermophilus HB8*	Batch culture	0.023 g/L/h	Diverse	Pantazaki et al., 2009
		*Methylobacterium* sp. *ZP24*	Batch culture	0.12 g/L/h	PHB	Yellore and Desai, 1998
Fermented whey supernatant	MMC	Undefined	Fed-batch culture	0.42 mg COD_PHA_ mg/COD_SS_	PHB/P-3(HB-co-HV)	Colombo et al., 2016
Fermented whey powder	MMC	Undefined	Fed-batch culture	0.68 gCOD/gCOD; 0.6 Cmol _PHA_/Cmol _Substrate_	P-3(HB-co-HV)	Gouveia et al., 2017; Fradinho et al., 2019
Fermented whey permeate + additives	MMC	Undefined	Fed-batch culture	0.59 gCOD_PHA_/gCOD_Substrate_	P-3(HB-co-HV)	Janarthanan et al., 2016
Hydrolyzed whey + additives	Pure cultures	*Halomonas halophila*	Batch cultures	0.05 g/L/h	PHB	Kucera et al., 2018
Whey/whey supernatant	Pure cultures	*Methylobacterium* sp. *ZP24*	Fed-batch culture	0.08 g/L/h	PHB	Nath et al., 2008
Hydrolyzed whey powder supernatant + additives	Pure cultures	*Haloferax mediterranei*	Batch culture	0.16 g/L/h	P(3HB-co-3HV)	Pais et al., 2016
Fermented whey powder supernatant + additives	MMC	Undefined	Batch culture	0.46 COD_PHA_/gCOD_Substrat_	P(3HB-co-3HV)	Valentino et al., 2015
Whey powder + additives	Engineered cultures	Engineered *Escherichia coli* with *Cupriavidus necator* PHA biosynthesis genes	Fed-batch culture	1.4 g/L/h	PHB	Wong and Lee, 1998

*^1^Values refer to the best yields obtained for PHA production from whey in each article. To facilitate comparison, when necessary, calculations were performed to arrive at values in g/L/h. Otherwise, we report the values as presented in the cited work. NS, not specified.*

Whey constitutes the main by-product of the dairy industry and is obtained by precipitation and removal of milk casein during cheese-making processes. Approximately 120 million tons of whey are produced annually throughout the world, of which only 50% are used for products used in human and animal feed (Nikodinovic-Runic et al., 2013). The remaining whey needs to be disposed of, which raises environmental concerns due to its relatively high organic load, as whey is mainly composed of lactose (39–60 g/L), fats (0.99–10.58 g/L), proteins (27–60 g/L), and mineral salts (4.6–8 g/L) (Prazeres et al., 2012; Colombo et al., 2016). From these cheese whey components, lactose is responsible for most of the biochemical oxygen demand of whey; therefore, it is essential to find a biotechnological use for lactose (Siso, 1996; Prazeres et al., 2012; Carvalho et al., 2013; Nikodinovic-Runic et al., 2013). The value of using whey lactose for PHA production is clear as it would dramatically decrease the costs of PHA production, without competing with the production of food for humans and simultaneously solving an environmental problem.

**FIGURE 1:**
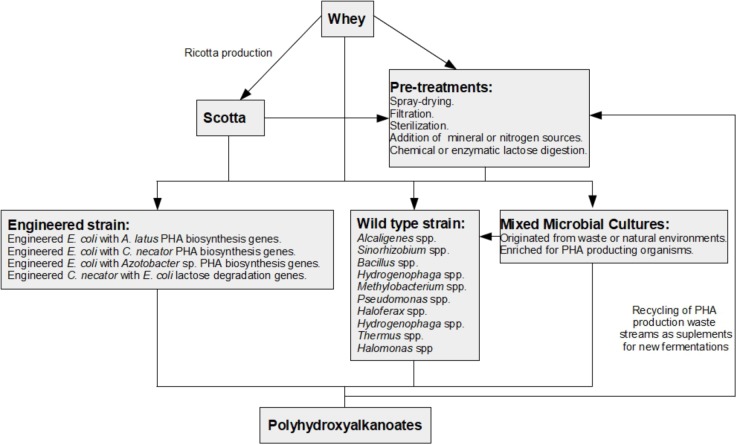
Schematic diagram of possible pathways for the production of polyhydroxyalkanoates from whey. Whole whey can be directly used in fermentations that produce PHAs. However, pre-treatments are normally used to generate efficient PHA production. Production of ricotta cheese from whey generates a by-product called ricotta cheese whey or *scotta*. *Scotta* can be used directly or after undergoing pre-treatments for PHA production. PHA production from whey can be achieved by using organisms that occur in nature and are capable of producing PHAs from whey or whey derivatives (wild-type strains); engineered strains enhanced for PHA production or lactose consumption (engineered strains); or mixed microbial cultures (MMCs) that can originate from waste streams or nature and can be enriched in PHA producing microorganisms usually using feast and famine cycles. During PHA production, waste streams are generated that can be used as additives for new fermentations from whey or *scotta*.

In this review, current knowledge on the use of cheese whey as a substrate for PHA production is summarized (Figure 1). Moreover, the problems and prospects of using whey as a substrate for industrial scale production of PHAs are pinpointed and discussed.

## Microorganisms Used for the Production of PHAs From Whey

Whey is a rich media that is clearly suited for microbial growth. However, finding microorganisms that efficiently produce PHAs when growing on whey has been shown to be extremely challenging. Unfortunately, some of the best-described PHA-producing microbial species have been shown to be unable to directly produce PHAs from whey. For instance, *Cupriavidus necator* [formerly known as *Ralstonia eutropha*, *Wautersia eutropha*, or *Alcaligenes eutrophus* (Vandamme and Coenye, 2004)] is capable of accumulating PHAs to up to 80% of its dry weight when growing on glucose (Ryu et al., 1997; Reinecke and Steinbüchel, 2008), but it is unable to efficiently grow and, consequently, produce PHAs on lactose, the predominant carbon source of whey (Povolo et al., 2010). Another example is the halophilic archaeal bacteria *Haloferax mediterranei*, which can accumulate PHAs to approximately 60% of its dry weight (Lillo and Rodriguez-Valera, 1990; Quillaguamán et al., 2010). Nevertheless, *H. mediterranei*, despite being capable of consuming lactose (Fernandez-Castillo et al., 1986), does not efficiently grow and produce PHA using this carbon source (Koller et al., 2007b). One exception is *Alcaligenes latus*, which is a known efficient PHA producer that can accumulate PHAs to up to 70% of its dry weight using diverse sugar sources (Yu et al., 1999; Gahlawat and Srivastava, 2017). Recently, one study showed that *Alcaligenes latus* is able to convert whey lactose into PHA (0.11 g L^-1^h^-1^) (Berwig et al., 2016) (Table 1). While the PHA yield obtained is not comparable to the yields obtained when *Alcaligenus latus* is grown in other carbon sources, more studies should be conducted to assess the full potential of using *A. latus* to produce PHAs from whey (Berwig et al., 2016).

The low capacity of highly PHA-producing microorganisms to produce PHA from whey lactose was, in some cases, bypassed by chemically or enzymatically converting whey lactose into glucose and galactose prior to fermentation. Using this hydrolyzed whey lactose, high PHA producers, such as *C. necator* and *H. mediterranei*, were shown to be able to produce PHAs (Marangoni et al., 2002; Pais et al., 2016) (Table 1). However, the steps required to convert whey lactose into glucose and galactose add to the final cost of PHAs and thus, from an industrial point of view, should be avoided. These conversion steps have, in fact, already been avoided by deploying genetic engineering techniques. For instance, *Escherichia coli* cells capable of consuming lactose were modified to express PHA biosynthesis genes from high PHA-producing microorganisms (Ahn et al., 2000, 2001). Alternatively, high producing strains were engineered to express lactose degradation genes (Povolo et al., 2010) (Table 1). While genetic engineering is a highly versatile and promising tool for enhancing PHA production from whey, the use of genetically engineered strains requires more controlled production plants. In fact, benefits and ethics aside, the wide application of genetic modified microorganisms (GMMs) in scientific research lab and biotechnology industry raises the concern of the accidental release of genetically modified species (or strains) into the environment and subsequent gene transfer to microorganisms/non-microorganisms host. In view of this, it has been considered necessary by biosafety regulations of individual countries to regulate their use avoiding any potential risks they may pose. The order requires notification, risk evaluation and approval of work with GMMs and approval of laboratories or plants where work with GMOs is to take place. Laboratories for working with GMMs are classified in four classes each with specific regulations with regard to containment, organization of the laboratories and the laboratory areas, plans for execution of the work, technical facilities, internal supervision, and control (Council Directive 98/81/EC of 26 October 1998 amending Directive 90/219/EEC on the contained use of genetically modified micro-organisms) (Devos et al., 2008; Guo et al., 2019). Therefore, biotechnology laboratories and industries require biosafety measures designed to protect their staff, the population, and the environment, which may be exposed to hazardous microorganisms and materials, as well as qualified staff, increasing the management costs of the plants and consequently the PHA production costs.

Considering all these observations, it is clear that an extremely valuable option for achieving economically competitive production of PHAs from whey would be to discover wild-type bacterial species that can directly produce high levels of PHA from whey lactose. However, currently, besides the abovementioned *A. latus* (Berwig et al., 2016), only a few wild-type microbes have been shown to be able to directly produce PHA from whey lactose and without exceptional PHA yields (Figure 1 and Table 1). Other microbes have been shown to produce PHAs from lactose-based media, including *Bacillus* sp. (Thirumala et al., 2010) and *Enterobacter cloacae* SU-1 (Samrot et al., 2011). Nevertheless, it remains unclear whether these microbes can use whey-derived lactose for PHA production.

Another option for whey-based PHA production that has recently received great attention is the use of mixed microbial cultures (MMCs) instead of single microbial strains (Colombo et al., 2016). While MMCs have been associated with lower yields of PHA production, they do not require sterile conditions and are able to adapt to changing industrial waste complex substrates (Reis et al., 2011; Colombo et al., 2016). MMCs have been used to directly produce PHAs from cheese whey lactose or after a first digestion of lactose by a different MMC (Carletto, 2014; Duque et al., 2014; Valentino et al., 2015; Colombo et al., 2016) (Table 1). MMCs are obtained from waste sources and are normally subsequently enriched for PHA production using feast and famine cycles designed to increase the presence of strains that are capable of creating PHA-based carbon reservoirs (Nielsen et al., 2017). Despite their great flexibility and cost advantages, using an unknown microbial community to produce PHAs can raise questions regarding the production efficiency, consistency of the polymer characteristics and biocompatibility when PHAs are to be used in medical applications. Nevertheless, the use of MMCs opens interesting prospects for identifying new PHA-producing microorganisms from these communities. Studies on MMCs showed that they consist of diverse bacterial genera, which change according to the tested fermentation carbon source (Janarthanan et al., 2016); however, a study in which single organisms were isolated for PHA production from whey has not, to our knowledge, been performed to date. Another possibility that would include the advantages of both MMCs and single strain fermentation would be to use MMCs to convert whey lactose into other carbon sources (such as organic acids) and then use a high PHA producer single strain to convert these organic acids into PHAs. This option has been proposed (Graziani and Fornasiero, 2007), but it has not yet been attempted for the production of PHAs from whey.

## Whey Pre-Treatments

Whey is a substrate that has numerous difficulties for direct use in PHA production. First, whey has been reported to have a low C-N rate, which greatly hinders PHA production (Bosco and Chiampo, 2010). Additionally, as previously discussed, lactose is not the preferred sugar of PHA-producing strains. Moreover, whey is a complex, unsterile and often variable by-product, and thus, its direct application in both lab-scale experiments and industrial production pipelines results in an abundance of other difficulties. Therefore, many studies that report PHA production from whey have actually been performed on whey derivatives that have undergone a series of pre-treatments (Figure 1 and Table 1) instead of whey itself. One of these derivatives is whey powder, which is diluted in water prior to fermentation to obtain a specific lactose concentration. Whey powder, normally produced by spray-drying, allows longer storage of whey as well as avoids seasonal variations of this by-product (Písecký, 2005; Chegini and Taheri, 2013). Nevertheless, a spray-drying step will increase costs in a PHA production pipeline. Thus, while acknowledging the possible consistency-based reasoning behind using whey powder for screening and optimizing PHA production, it remains unclear whether fermentations using whey are comparable to those performed with a whey powder.

The other extensively used pre-treatment of whey, regardless of whether it is a liquid or powder, is the removal of the majority of its proteins and other solids by ultrafiltration, creating a whey permeate that retains most of the whey lactose (Koller et al., 2007b). In addition to ultrafiltration, this protein removal is also commonly obtained by acidifying whey to a pH close to 4 followed by a heat treatment, centrifugation and filtration, generating a so-called whey supernatant (Pantazaki et al., 2009). These whey permeates or supernatants are easier to work with, as they are sterile, homogeneous and clear solutions; however, their use increases the production costs. Moreover, it was shown that whole whey and whey supernatant, used as carbon sources in a complex mineral media, led to different biomass growth and PHA accumulation in *Methylobacterium* sp. *ZP24* (Yellore and Desai, 1998). Therefore, attention should be paid when extrapolating results obtained from whey permeates or supernatants to whole whey.

Other types of additives, which are routinely used when dealing with whey in PHA production studies, consist of minerals and nitrogen sources (Table 1). The use of these additives could probably be avoided by using whole whey instead of whey permeates or supernatants. However, for some studies, it remains to be seen whether the used microorganisms are capable of accumulating high amounts of PHAs using whey without additives, which would be ideal for industrial production. For *Bacillus megaterium*, the addition of salts ((NH_4_)_2_SO_4_, 5 g L^-1^; Na_2_HPO_4_, 2.5 g L^-1^; KH_2_PO_4_, 2.5 g L^-1^; MgSO_4_, 0.2 g L^-1^; and MnSO_4_, 0.01 g L^-1^) to whey supernatant increased its biomass and PHA production by almost 10 times (Obruca et al., 2011). Therefore, if any addition should be made to whey for enhanced PHA production, it would be interesting to explore the use of other inexpensive waste products as whey additives; this point will be discussed subsequently in this paper.

Whole whey should be more widely used to study PHA production. However, the use of whole whey raises important experimental challenges, including sterility issues. These issues are not as relevant when using MMCs; however, for single cultures, sterilization is crucial (Koller et al., 2011). Sterilization of whole whey with high temperatures cannot be performed as total whey protein will precipitate, and filtration is difficult due to the high quantity of suspended solids present in whey. Low-temperature pasteurization could be used, but it normally involves several cycles of heat and cold treatments that result in expensive and time-consuming processes (Ghaly and Kamal, 2004). Moreover, pasteurization does not assure full sterilization, which can lead to sterility problems during fermentation processes. Therefore, alternative sterilization techniques, such as the use of ultraviolet radiation or high temperature-short time pasteurization, have been proposed to be used prior to fermentation with pure strains in whole whey (Morr, 1987; Ghaly and Kamal, 2004). Furthermore, the use of antibiotics has also been attempted to solve these issues. Vancomycin was shown to reduce *Bacillus cereus* contamination in *Hydrogenophaga pseudoflava* PHA production from whey without significantly affecting the PHA production efficiency (Koller et al., 2011). Another problem that arises by using untreated whole whey is the high lactose concentration. It was shown that the whey lactose concentration was crucial for biomass and PHA production by *B. megaterium* CCM 2037. In fact, while growing on whey supernatant with a lactose concentration of 40 g/L, this strain grew to approximately 1.5 g/L biomass and produced less than 0.5 g/L PHAs; when grown in a diluted whey supernatant with a concentration of 20 g/L, the production values increased to 2.51 g/L biomass and produced 0.79 g/L PHAs (Obruca et al., 2011).

In addition to these whey pre-treatments, for specific microorganisms, as previously mentioned, transformation of whey lactose into galactose and glucose needs to be performed prior to fermentation (Table 1). Some authors have avoided lactose hydrolysis by using media that mimics hydrolyzed whey containing galactose and glucose as carbon sources (Wallner et al., 2001; Koller et al., 2011). The majority of the remaining studies proceeded to hydrolysis using β-galactosidase enzymes and showed that microbes were able to use glucose and galactose originating from enzymatic hydrolysis of cheese whey lactose (Marangoni et al., 2002; Koller et al., 2005, 2007a,b, 2008; Koller, 2015). However, the use of enzymatic hydrolysis greatly increases the costs of a possible PHA production plan. Thus, it is necessary to highlight that it has also been shown that chemical lactose hydrolysis using HCl resulted in whey lactose hydrolysates that could be used for microbial production of PHAs (Pais et al., 2016; Kucera et al., 2018). Nevertheless, preferably, these treatments should be avoided by finding either wild-type microorganisms that accumulate high levels of PHAs using lactose as a carbon source or microorganisms that convert lactose into more widely used carbon sources for PHA production.

The use of MMCs avoids most of these problems, as, in theory, a community is able to thrive and evolve in diverse environments and still produce PHAs. However, this hypothesis has not been widely tested, as most studies used whey powder derivatives to test MMC production of PHAs (Table 1). In addition to whey powder, whey permeate has also been used (Carletto, 2014) as well as the supernatant of whey, which has been subjected to a first fermentation to transform whey lactose (Colombo et al., 2016). Interestingly, the use of these whey supernatants yielded more PHA production than those obtained by using whey powder and whey permeates (Colombo et al., 2016). These results led us to believe that the use of MMCs may be the correct approach for producing PHAs from whole whey. However, these results also suggest that there are considerable PHA productivity differences when using diverse whey derivatives. Therefore, MMCs should be tested for PHA production using whole whey.

Finally, most of the current knowledge on using whey for PHA production is based on studies that use whey derivatives to ensure experimental consistency and ease (Table 1). However, it is now time to use whole whey, as this is crucial for obtaining a comprehensive view of the actual feasibility of using whey for PHA production in an industrially viable manner.

## Alternative Routes to Improve PHA Production From Whey

In this review, the focus is on two important factors that need to be taken into consideration when aiming at achieving an economically viable production of PHA from cheese whey: microorganism choice and whey pre-treatment requirements. While acknowledging that not all factors can be reviewed here, it is necessary to mention, nonetheless, that some other important aspects need to be considered to achieve efficient production of PHAs from whey.

### Recycling in PHA Production From Whey

As discussed in the previous sections, to achieve efficient production of PHAs from whey, it is necessary to use several additives to whey. Thus, it would be extremely interesting if these additives could originate from other waste streams. Fermentations of enzymatically digested whey lactose for PHA production by *H. mediterranei* were used to test the feasibility of using recycling waste streams in future PHA production pipelines (Koller, 2015). *H. mediterranei* is an extreme halophile, and as a consequence, its use has the advantages of reducing sterilization costs and enabling easier PHA extraction methods based on differential osmotic pressures (Koller et al., 2007b). Nevertheless, fermentations for PHA production using *H. mediterranei* generate vast quantities of highly saline waste streams. Therefore, authors have assessed the possibility of using these obtained waste streams in subsequent fermentations. Despite the low yields obtained, the re-use of waste streams allowed PHA production, opening exciting prospects for further studies on re-using waste streams in PHA production processes (Koller, 2015). Another interesting prospect that should be explored and that, to our knowledge, is still undescribed is to use these streams in other biotechnological production processes. Therefore, studies that investigate the use of recycling waste streams during PHA production from whey would be of extreme significance when aiming to create added value using environmentally friendly industrial processes.

To achieve optimized PHA production from whey, it has been shown that the use of additives, such as salts or nitrogen sources, is required. Thus, and as mentioned previously, it would be of extreme value if these additives could derive from other food wastes. While this type of additive source for PHA production from whey remains greatly unexplored, whey has already been used as an additive for PHA production using waste frying oils. Protease hydrolyzed whey has been shown to work as a complex nitrogen source to enhance PHA production from waste frying oils using *C. necator* by approximately 40% (Obruca et al., 2014). Therefore, the mixing of waste sources opens nearly endless possibilities to enhance the efficiency of PHA production without the need for costly additives. In addition to other waste streams, low cost additives could also be tested for PHA production enhancements. The addition of 1% ethanol as an exogenous stress factor on cheese whey, for instance, was shown to enhance *B. megaterium* PHA production by approximately 40% (Obruca et al., 2011). The use of ethanol or other stress factors, such as PHA production enhancers, should definitively be taken into account when optimizing PHA production from cheese whey.

As previously stated, whole whey is a complex and rich media that can create obstacles to sterilization, fermentation efficiency and PHA extraction methods. Thus, whey pre-treatments are normally required and have an impact on production costs. It would be helpful if whole whey could be first used in a different biotechnological process, followed by the use of the residues of this process for PHA production. Whole whey is commonly used in *ricotta* cheese-making, which generates a by-product called ricotta cheese whey or *scotta* (Sansonetti et al., 2009; Bergamaschi and Bittante, 2018). *Scotta* retains most of the lactose from whey but has a highly reduced protein content (De Giorgi et al., 2018), which could initially inhibit microbial growth and interfere with PHA extraction processes. The production of *scotta* mostly occurs in Italy, where it accounted for approximately one million tons in 2009 and has already been tested as a carbon source for several biotechnological processes, such as the production of bioethanol (Zoppellari and Bardi, 2013), biodiesel (Carota et al., 2017), probiotics (Lavari et al., 2014), lactic acid (Secchi et al., 2012), and lactobionic acid (De Giorgi et al., 2018). It has also been shown that *scotta* can possibly be used to produce PHA via fermentation by MMCs. MMCs were demonstrated to be able to produce PHA from *scotta* without any pre-treatment other than a filtration step and achieve PHA productivities of up to 1.65 g/L (Carletto, 2014). Thus, *scotta* production could prove to be a productive and extremely unexplored middle step in the efficient transformation of cheese whey into PHAs. Nonetheless, further studies are needed to determine the full potential of using *scotta* in industrial scale PHA production.

### PHA Purification From Whey

Another possible problem that can arise from using whole whey for microbial PHA production is connected to the extraction and purification of PHAs. Initially, there were mainly two strategies to extract PHAs from bacterial cells. The first strategy used solvents, such as chloroform and methanol, taking advantage of the solubility of PHA in chloroform and its insolubility in methanol (Byrom, 1987; Suriyamongkol et al., 2007). With these solvent-based methods, high yields of PHA extraction were obtained (greater than 90%). However, the use of solvents makes these processes economically as well as environmentally unappealing (Byrom, 1987). The second strategy involved the use of enzymes and detergents that remove cellular components while leaving PHAs intact (Suriyamongkol et al., 2007). These strategies are more environmentally friendly but normally result in lower PHA yields and, when using enzymes, also high costs (Mozejko-Ciesielska and Kiewisz, 2016). Several new solvents and methods are being tested to substitute for the more widely used extraction methods (Madkour et al., 2013), including the prospect of creating microbial strains that are capable of secreting PHAs (Sabirova et al., 2006). Despite interest in this strategy, it has not been followed up, with the exception of a study that showed partial PHA secretion from engineered *E. coli* cells (Rahman et al., 2013). Nevertheless, for PHAs production from whey, there has not been a deep investigation into alternative and cheaper PHA extraction methods. The only alternative environmentally friendly method tested for PHA extraction from whey works solely with one microorganism because it takes advantage of the inner high osmotic pressure of *H. mediterranei* cells to easily achieve cell disruption (Koller, 2015). Moreover, as previously stated, most studies used whey permeates and supernatants as substrates for PHA production. It, therefore, remains to be shown whether the extraction methods used result in comparable PHA recovery yields and purities when using whole whey, which contains large amounts of proteins and lipids and creates another level of complexity for PHA extraction processes.

### Type of PHAs Produced From Whey

The types of hydroxyalkanoate (HA) monomers that are being produced constitute another factor that needs to be carefully considered when analyzing PHA production costs, as there have been more than 150 HA monomer types identified (Reis et al., 2011). PHAs with different monomer compositions and structures present diverse economically important properties that affect their market value. The most common HA monomer produced by bacteria is 3-hydroxybutyrate (3HB), and PHAs composed of only this monomer have economically interesting properties. Nonetheless, PHAs that incorporate other monomers different from 3HB in their chains, such as 3-hydroxyvalerate (3HV), have generally improved market desired properties (Reis et al., 2011; Mozejko-Ciesielska and Kiewisz, 2016; Albuquerque and Malafaia, 2018). Therefore, it is important to consider the PHA monomer composition while optimizing PHA production from whey as, recently, different whey pre-treatments were shown to lead to the production of different types of PHAs. Cheese whey fermented by autochthonous cheese lactic acid bacteria and subsequently used for PHA production by an MMC led to the production of PHAs composed solely of 3HB monomers. On the other hand, MMCs produced PHA co-polymers composed of both 3HB (60%) and 3HV (40%) monomers when using cheese whey fermented by inoculated sporogeneous acidogenic bacteria. These results are explained by the presence of several HB precursors (including lactic, butyric, and acetic acids) in fermented cheese whey obtained by the action of autochthonous cheese lactic acid bacteria, in contrast to the presence of HV precursors (for instance, propionic and valeric acids) in cheese whey fermented by inoculated sporogeneous acidogenic bacteria (Colombo et al., 2016). These results demonstrate that whey is a suitable carbon source for the production of high-value PHAs and also the importance of testing whole whey for PHA production because changes in fermentation substrates influence not only the PHA yield but also its monomer composition.

## Conclusion

Economically and environmentally viable production of bioplastics is required to solve the environmental problems that arise from the production and accumulation of conventional plastics. PHAs could play an important role in this bioplastic solution because they have market-desired properties and can be biologically produced from inexpensive waste substrates, such as whey. However, there are still numerous challenges that need to be overcome to achieve efficient production of PHAs from whey. In this work, the studies that describe PHA production from whey focusing on whey pre-treatments and the choice of producing microorganisms have been summarized (Table 1). Our current knowledge on PHA production from whey provides clear indications that producing PHA from whey is possible and can be achieved using diverse strategies. Notwithstanding, this review also highlights that there is a shortage of studies that aim at using whey to produce PHAs at a large scale and consider the costs and environmental impacts of future industrial producing plants. Therefore, this study could motivate and guide future studies focusing on obtaining economically viable production of PHAs from whey.

## Author Contributions

TA was responsible for data collection, wrote the first draft of the manuscript, and revised the article. DR and GC revised the manuscript. LI was responsible for the conception and design of the manuscript and revised it critically.

## Conflict of Interest Statement

The authors declare that the research was conducted in the absence of any commercial or financial relationships that could be construed as a potential conflict of interest.

## Footnotes

1 European bioplastics: Available online at: https://www.european-bioplastics.org/bioplastics/materials/; accessed on 20/02/2018.

2 European bioplastics: Available online at: https://www.european-bioplastics.org/market/; accessed on 20/02/2018.
